# *Caspase-1* variant influencing CSF tau and FDG PET levels in non-demented elders from the ADNI cohort

**DOI:** 10.1186/s12883-022-02582-9

**Published:** 2022-02-16

**Authors:** Yi Liu, Meng-Shan Tan, Zuo-Teng Wang, Wei Xu, Lan Tan

**Affiliations:** Department of Neurology, Qingdao Municipal Hospital, Qingdao University, Qingdao, China

**Keywords:** *Caspase-1*, Variant, CSF, Tau pathology, FDG PET, Neurodegeneration, ADNI

## Abstract

**Background:**

Genetic variations in the inflammatory *Caspase-1* gene have been shown associated with cognitive function in elderly individuals and in predisposition to Alzheimer’s disease (AD), but its detailed mechanism before the typical AD onset was still unclear. Our current study evaluated the impact of *Caspase-1* common variant rs554344 on the pathological processes of brain amyloidosis, tauopathy, and neurodegeneration.

**Methods:**

Data used in our study were obtained from the Alzheimer’s Disease Neuroimaging Initiative (ADNI) cohort. We examined the relationship between *Caspase-1* rs554344 allele carrier status with AD-related cerebrospinal fluid (CSF), PET, and MRI measures at baseline by using a multiple linear regression model. We also analyzed the longitudinal effects of this variant on the change rates of CSF biomarkers and imaging data using a mixed effect model.

**Results:**

We found that *Caspase-1* variant was significantly associated with FDG PET levels and CSF t-tau levels at baseline in total non-demented elderly group, and especially in mild cognitive impairment (MCI) subgroup. In addition, this variant was also detected associated with CSF p-tau levels in MCI subgroup. The mediation analysis showed that CSF p-tau partially mediated the association between *Caspase-1* variant and CSF t-tau levels, accounting for 80% of the total effect.

**Conclusions:**

Our study indicated a potential role of *Caspase-1* variant in influencing cognitive function might through changing tau related-neurodegeneration process.

**Supplementary Information:**

The online version contains supplementary material available at 10.1186/s12883-022-02582-9.

## Background

Immune system-mediated inflammation exerts an enormous function on neurodegenerative diseases, particularly in Alzheimer’s disease (AD) [1, 2]. Recently, increasing numbers of researches have indicated that inflammasome, as an indispensable part of the innate immune system, which activates downstream inflammatory cytokines by Caspase-1, plays a significant role in AD pathogenesis [3–7]. Caspase-1, also referred to as interleukin-1β(IL-1β)-converting enzyme, could cleave the precursors of inflammatory cytokines into their active forms [8]. Furthermore, Caspase-1 could incise gasdermin D and thus cause pyroptotic cell death, promoting the inflammatory cytokines to secrete outside the cell [9]. What’s more, genetic studies found that variations (rs554344, rs580253) in the *Caspase-1* gene were associated with cognitive function in elderly individuals with normal cognition [10], and in progression from mild cognitive impairment (MCI) to AD [11], but its detailed mechanism before the typical AD onset was still unclear till now.

As studies have shown, patients could experience a long period of time before developing into AD, accompanied by changes in cerebrospinal fluid (CSF) biomarkers and imaging data [12]. Recently, the National Institute on Aging and Alzheimer’s Association (NIA-AA) updated [13]. The new guidelines, better known as the NIA-AA research framework, could be used for observational and interventional research, which defines AD by three biomarkers in living person: β-amyloid (Aβ) deposition, including amyloid-PET, CSF Aβ_42_ or Aβ_42_/Aβ_40_ ratio; pathologic tau, including tau-PET, CSF phosphorylated tau (p-tau); Neurodegeneration, including fluorodeoxyglucose (FDG) PET, CSF total tau (t-tau) or brain structural MRI.

Hence, our current study examined the impact of *Caspase-1* variant rs554344 on the pathological processes of brain amyloidosis, tauopathy, and neurodegeneration, using the baseline and follow-up data from AD-related CSF, PET, and MRI measures in a large non-demented population, including normal cognition (NC) and MCI subgroups, from the Alzheimer’s Disease Neuroimaging Initiative (ADNI) database.

## Methods

### ADNI database and participants

The data used in our analysis were obtained from the ADNI database (http://adni.loni.usc.edu). ADNI was launched in 2003 as a public-private partnership, led by Principal Investigator Michael W. Weiner, MD, VA Medical Center and University of California-San Francisco (www.loni.ucla.edu/ADNI), which provide clinical, imaging, genetic, and biochemical information for AD research. For more information, see www.adni-info.org [14].

Here, we restricted our present analysis to MCI and NC subjects whose genotype data of *Caspase-1* rs554344 were available. It is noteworthy that rs554344 and rs580253 are in linkage disequilibrium (r^2^ = 1) and occur together in one haploblock [10]. Furthermore, we selected only non-Hispanic white individuals in order to avoid the effects of population stratification which can lead to spurious findings. Finally, 698 non-demented elderly individuals including 442 MCI and 256 NC at baseline were included in our study (Table 1).

**Table 1:** Demographic and clinical characteristics of included subjects in ADNI

Characteristics	Non-demented elderly		MCI		NC		***P***-value^*^
Age (n, means±SD)	698	73.61 ± 6.75	442	72.54 ± 7.38	256	74.80 ± 5.40	< 0.001
Gender (n, male/female)	698	395/303	442	266/176	256	129/127	0.012
Education (n, means±SD)	698	16.05 ± 2.82	442	15.97 ± 2.85	256	16.43 ± 2.66	0.068
*APOE ε4* (n, 0/1/2 alleles)	698	428/225/45	442	240/163/39	256	188/62/6	< 0.001
MMSE (n, means±SD)	698	28.33 ± 1.61	442	27.5 ± 3.46	256	28.12 ± 5.10	< 0.001
*Caspase-1* rs554344 (n, GG/GC/CC)	698	476/198/24	442	305/127/10	256	171/71/14	0.393
AV45 PET (n, means±SD)	426	1.18 ± 0.21	290	1.21 ± 0.22	136	1.12 ± 0.18	< 0.001
FDG PET (n, means±SD)	555	1.27 ± 0.12	366	1.25 ± 0.13	189	1.30 ± 0.11	< 0.001
CSF- Aβ_42_ (n, means±SD)	410	918.60 ± 365.61	282	871.38 ± 349.02	128	1022.61 ± 380.92	< 0.001
CSF t-tau (n, means±SD)	516	262.78 ± 100.61	334	274.15 ± 109.25	182	242.60 ± 81.37	< 0.001
CSF p-tau (n, means±SD)	515	24.63 ± 10.79	333	26.80 ± 11.93	182	22.07 ± 8.08	< 0.001
HVa (n,means±SD)	620	7055.30 ± 1023.75	387	6901.99 ± 1099.49	233	7309.94 ± 825.04	< 0.001

*Abbreviation*: *Aβ*_*42*_ amyloid-β, *CSF* cerebrospinal fluid, *HVa* adjusted hippocampal volume, *MCI* mild cognitive impairment, *MMSE* Mini-Mental State Exam, *N* number, *NC* normal cognition, *p-tau* phosphorylated tau, *SD* standard deviation, *t-tau* total tau

Notes: *P-*value^*^ represents the difference between MCI and NC groups

### CSF biomarker data

CSF biomarker data, including CSF Aβ_42_, CSF t-tau, and CSF p-tau, was acquired from ADNI database. The collection and manipulation of CSF data have been mentioned in preceding study [15]. These data were computed using the xMAP Luminex platform with Innogenetics/Fujirebio AlzBio3 immunoassay kits at the ADNI Biomarker Core Laboratory (University of Pennsylvania). All CSF biomarker assays were performed in duplicate and averaged as previously described [16].

### PET data acquisition and analyses

A detailed description of PET image acquisition and processing can be found at http://adni.loni.usc.edu/datasamples/pet/. The PET data was obtained from UC Berkeley and the Jagust Lab on the website (http://adni.loni.usc.edu/data-samples/access-data/). The AV45-PET (amyloid-PET) standardized uptake value ratios (SUVRs) were formed by normalizing composite multi-region target regions of interest (ROIs) to the cerebellar crus gray matter as previously described [17]. In addition, mean FDG uptake was averaged from 5 meta-ROIs including right and left angular gyri, right and left inferior temporal regions, and bilateral posterior cingulate. We intensity-normalized each meta-ROI mean by dividing it by the pons/vermis reference region mean [18].

### Structural MRI data

Hippocampal volume (HV) and estimated intracranial volume (eICV) used in the ADNI subjects were acquired from UCSF data in ADNI dataset by a Siemens Trio 3.0 T or 1.5 T scanner. The method has been described in details [19]. We selected the brain area - hippocampus as region of interest, which are critical regions for AD pathology. The following formula was used to calibrate hippocampal volume: Adjusted HV (HVa) = Raw HV – b (eICV – Mean eICV), where b is the coefficient when HV is regressed relative to eICV [17].

### Statistical analysis

All statistical analyses were carried out through R 4.02 and PLINK 1.07. First, we examined the relationship between *Caspase-1* rs554344 allele carrier status with AD-related phenotypes at baseline by using multiple linear regression model. Since the minor allele homozygote (CC) frequency of rs554344 was less than 2%, we used a dominant genetic model to code *Caspase-1* genotype as 0 and 1, which means whether or not they carry the C alleles. Then, basing on the methods proposed by Baron and Kenny [20], we conducted parametric mediation analysis to estimate the effect of *Caspase-1* rs554344 on CSF t-tau mediated through CSF p-tau, or on FDG PET mediated through CSF p-tau or t-tau. In this mediation analysis, the effect was defined in terms of the difference in regression coefficient by logistic regression models. Finally, over a mean follow-up of 3 years, range from 0.25 to 15 years, the association of *Caspase-1* rs554344 with longitudinal CSF biomarkers and imaging data was analyzed by a mixed effects model in order to obtain the trend of the variable as time goes on. Follow-up information was available for 698 non-demented elderly individuals. In all of the above analyses, age, gender, education years and *APOE ε4* status were taken as covariates for test. The *APOE* genotype was coded as 0, 1, and 2 to present owning 0, 1, and 2 *ε4* alleles, respectively. The FDG PET levels fitted the normal distribution, and the data were all checked for normality. After log transformation, t-tau and p-tau levels also fitted the normal distribution. If *p* <  0.05, we considered the results are statistically significant.

## Results

The demographic information of the included subjects was shown in Table 1. In details, 698 non-dementia individuals (303 women, 73.61 ± 6.75 years), including 256 NC subjects (127 women, 74.80 ± 5.40 years) and 442 MCI subjects (176 women, 72.54 ± 7.38 years), were recruited in this study. No statistical differences were observed between NC and MCI when comparing the distribution of the allele frequencies of rs554344 in our study. In addition, there was no demographic difference between *Caspase-1* allele carriers (GG subjects and GC + CC subjects). As expected, the two subgroups had statistically significance in Mini-Mental State Examination (MMSE) scores and *APOE ε4* allele frequency (*p* <  0.001). The MCI group had significantly smaller volumes in HVa (*p* <  0.001), and higher levels of CSF t-tau (*p* <  0.001), p-tau (*p* <  0.001) and AV45 PET (*p* <  0.001), while lower levels of CSF Aβ (*p* <  0.001) and FDG PET (*p* <  0.001) when compared to those of the NC group.

**Fig. 1:**
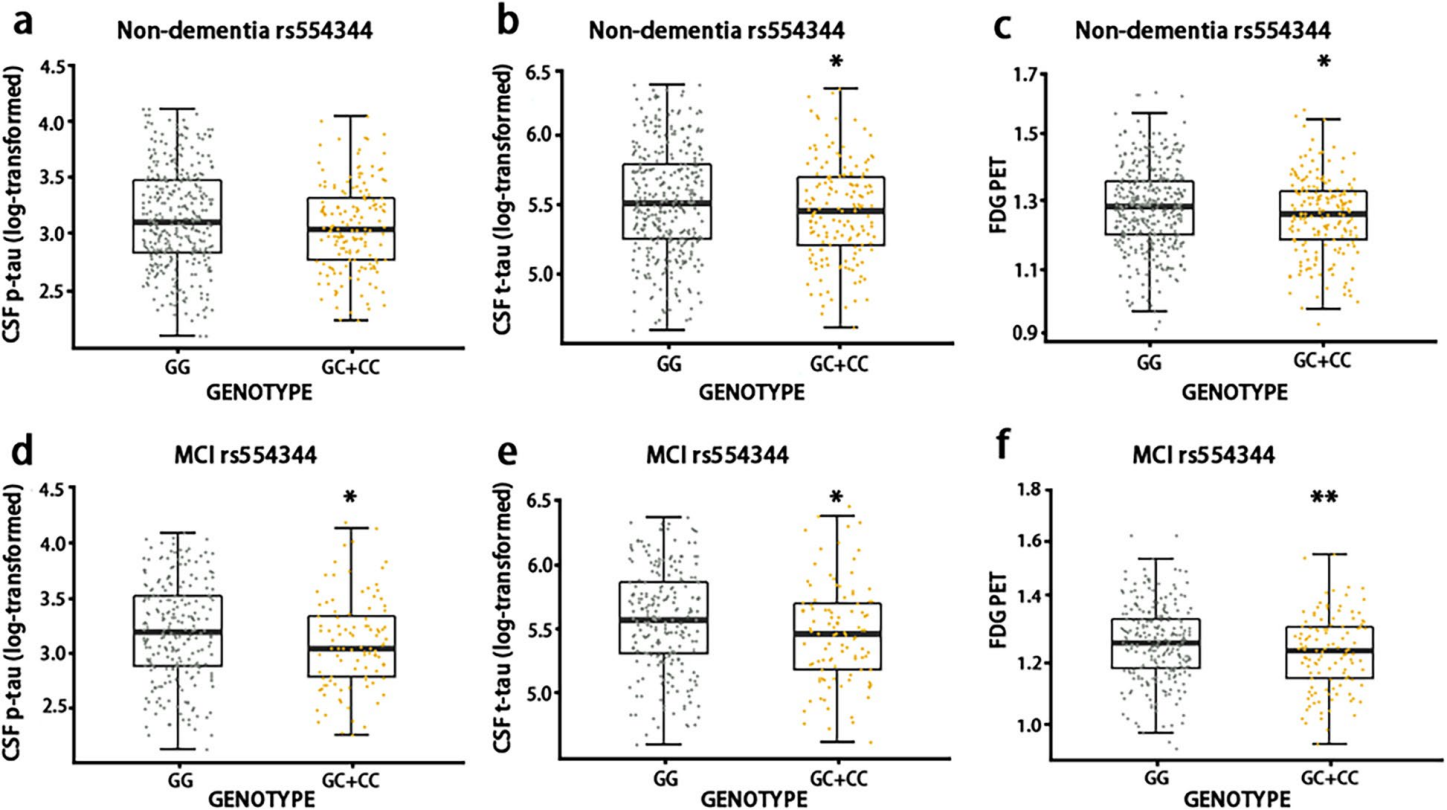
The frequency distribution of *Caspase-1* rs554344 at CSF p-tau, t-tau, and FDG PET levels in the boxplots. *Caspase-1* variant rs554334 was significantly related with CSF t-tau and FDG PET levels at baseline in the total non-demented elderly group and MCI subgroup (**b**,**c**,**e**,**f**). Besides, *Caspase-1* rs554344 was found the trend related to CSF p-tau levels at baseline in the total non-demented elderly group, but it didn’t reach statistical significance (**a**). In MCI subgroup, *Caspase-1* rs554334 showed statistical significance with CSF p-tau levels (**d**). ^*^p < 0.05, ^**^p < 0.01. Notes: Our data about t-tau, p-tau levels fitted the normal distribution after log transformation

**Fig. 2:**
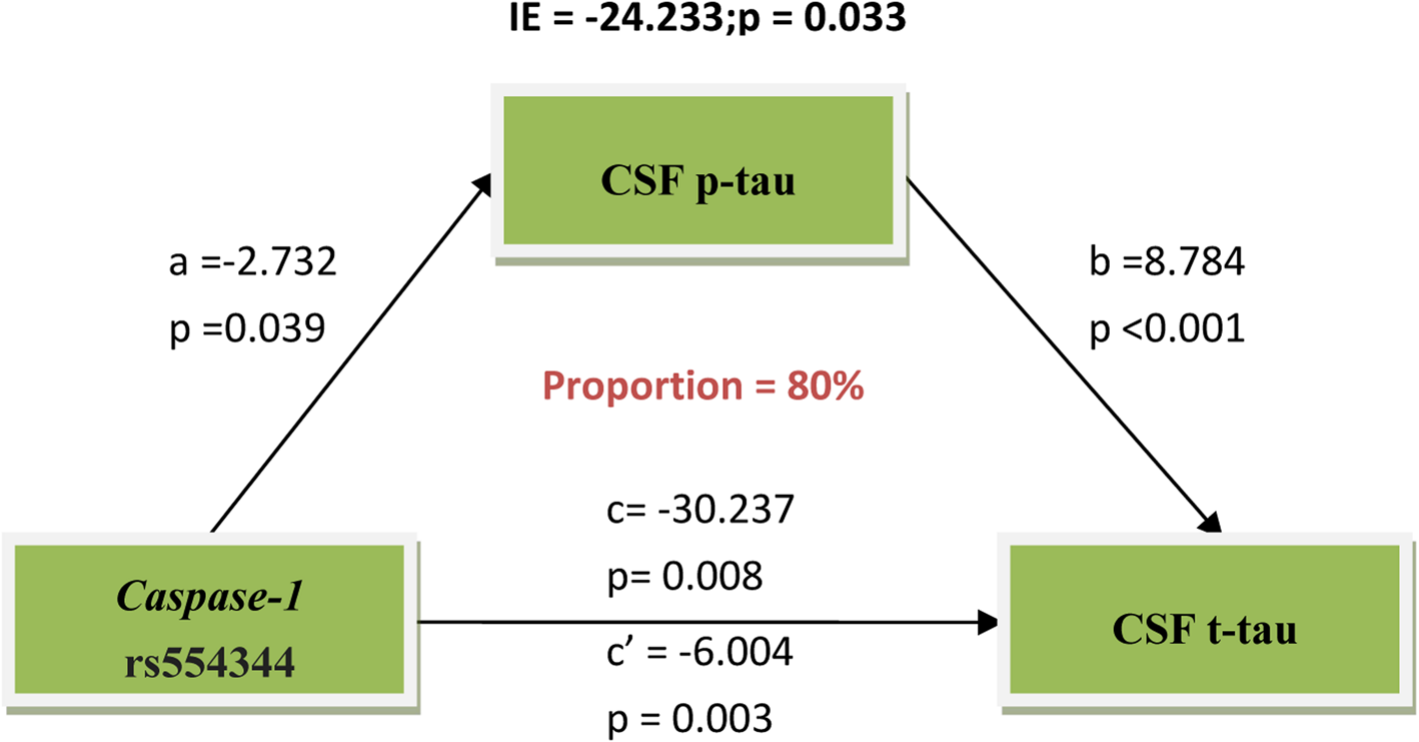
The relationship between *Caspase-1* rs554344 and CSF t-tau levels was mediated by CSF p-tau. The total effect of *Caspase-1* rs554344 on CSF t-tau was estimated and was divided into direct effect and the mediated effect through CSF p-tau. The mediation analysis showed that CSF p-tau significantly and partially mediated the association between *Caspase-1* variant and CSF t-tau levels, accounting for 80% of the total effect

In our current study, we first analyzed the relationship between *Caspase-1* rs554344 with AD-related phenotypes, including CSF biomarkers and image data at baseline (Table 2). Our results demonstrated that *Caspase-1* loci genotype was significantly associated with CSF t-tau (β Coefficient and 95% confidence interval (CI) = − 0.068 (− 0.134 – − 0.002), *p* <  0.05) and FDG PET levels (β Coefficient and 95% CI = − 0.026 (− 0.46 – − 0.06), *p* <  0.05) at baseline in the total non-demented group, and further subgroup analysis shown the above positive findings were also in MCI subgroup (β Coefficient and 95% CI = − 0.100 (− 0.186 – − 0.015), *p* <  0.05 for CSF t-tau; β Coefficient and 95% CI = − 0.034 (− 0.060 – − 0.008), *p* <  0.05 for FDG PET levels). Besides, in MCI subgroup, *Caspase-1* loci genotype showed statistical significance with CSF p-tau levels (β Coefficient and 95% CI = − 0.098 (− 0.195 – − 0.001), *p* <  0.05) (Fig. 1). In addition, age and *APOE ε4* status (β Coefficient and 95% CI = − 0.003 (− 0.005 – − 0.002), *p* <  0.001 for age; β Coefficient and 95% CI = − 0.049 (− 0.067 – − 0.032), *p* <  0.001 for *APOE ε4* status) were closely correlated with CSF biomarkers, image data among covariates (Table S1). The above findings suggested that *Caspase-1* variant influences cognition via CSF p-tau, CSF t-tau, and FDG PET, but it’s not clear whether the two of them are related. CSF p-tau represents an aberrant pathologic condition linked with PHF tau accumulation, whereas CSF t-tau and FDG PET represent the degree of neuronal damage [13]. Then, we conducted parametric mediation analysis to estimate the effect of *Caspase-1* rs554344 on CSF t-tau and FDG PET. Our mediation analysis showed that CSF p-tau significantly and partially mediated the association between *Caspase-1* variant and CSF t-tau levels (Fig. 2). The effect was considered partial mediation with the proportion of 80%. However, *Caspase-1* acting on FDG PET did not mediate through CSF t-tau or CSF p-tau (Fig. S1). While, in our current longitudinal analysis, there was no statistical evidence for an effect of rs554344 allele carrier status on longitudinal CSF biomarkers and image data in total non-demented group after controlling for age, gender, education and *APOE ε4* status (Table S2).

**Table 2:** The correlation between *Caspase-1* rs554344 and CSF biomarkers, imaging data at baseline in a multiple linear regression model

Characteristics	N	Effect	CSF-Aβ_**42**_ (pg/ml)	CSF t-tau (pg/ml)	CSF p-tau (pg/ml)	FDG PET (SUVRs)	AV45 PET (SUVRs)	HVa (mm^3^)
Non-demented elderly	698	β Coefficient	−0.217	− 0.068	−0.069	− 0.026	−0.005	118,898
		*P*-value	0.851	**0.043**	0.066	**0.013**	0.623	0.829
MCI	442	β Coefficient	−0.068	−0.100	−0.098	− 0.034	0.001	−3.196
		*P*-value	0.146	**0.021**	**0.049**	**0.009**	0.977	0.976
NC	256	β Coefficient	93.583	0.038	0.042	−0.018	0.005	−32.772
		*P*-value	0.171	0.449	0.434	0.278	0.756	0.760

*Abbreviation*: *Aβ*_*42*_ amyloid-β, *CSF* cerebrospinal fluid, *HVa* adjusted hippocampal volume, *MCI* mild cognitive impairment, *N* number, *NC* normal cognition, *p-tau* phosphorylated tau, *t-tau* total tau

## Discussion

AD is an intractable progressive neurodegenerative disease characterized by cognitive decline and dementia. An inflammatory neurodegenerative pathway, involving Caspase-1 activation, is associated with human age-dependent cognitive impairment and several classical AD brain pathologies. Previous studies have shown that Aβ can activate inflammasome and Caspase-1 [21, 22], further mediates memory loss [23, 24]. However, before the typical AD onset, the detailed mechanism by which Caspase-1-dependent inflammation leads to cognitive decline remains undefined.

In this study, for the first time, we investigate the association between *Caspase-1* rs554344 with CSF and imaging biomarkers from the ADNI database. We found that *Caspase-1* variant was significantly associated with FDG PET levels and CSF t-tau levels at baseline in total non-demented elderly group. What’s more, in MCI subgroup, we discovered that 1) the correlation between *Caspase-1* variant and CSF p-tau levels; 2) the influence of *Caspase-1* variant on CSF t-tau levels was mainly mediated by CSF p-tau. These findings indicated a potential role of *Caspase-1* variant in influencing tau related-neurodegeneration process. Previous study has also supported that inflammasome activation could drive tau pathology process [4].

According to the NIA-AA research framework in 2018, we know that FDG PET and CSF t-tau are biomarkers of Neurodegeneration, while CSF p-tau is indicator of aggregated Tau pathology [13]. The combination of neuronal injury, aggregated tau and aggregated Aβ can provide a strong basis for the progression of AD. Nevertheless, both in the MCI and NC subgroup, we did not find any statistical significance between *Caspase-1* rs554344 and Aβ deposition biomarkers. This does not prevent Caspase-1 to becoming target for tau related-neurodegeneration diseases therapy, because the tau pathology plays an increasingly important role in AD pathogenesis [25]. Despite the distribution of the *Caspase-1* rs554344 frequency between NC and MCI being similar, our current results support the role of *Caspase-1* rs554344 in AD tauopathy. With the addition of the ADNI database, there will be a larger sample size to support our views in the future. Besides, a negative correlation between p-tau and cognitive functions was previously observed in patients with neurodegenerative tauopathy [26]. It has also been proved that inhibitors of Caspase-1 can alleviate cognitive impairment of AD model mouse [24, 27, 28]. Given the above evidences, we have reason to speculate the Caspase-1-induced spatial cognitive deficits might through changing tau related-neurodegeneration process. Therefore, modulating the activation of Caspase-1 may be a potential therapeutic strategy for neurodegenerative tauopathies. And, inhibitors of Caspase-1 might have the capacity to arrest or delay the progress of neurodegeneration in the future.

## Conclusions

To sum up, our study indicated a potential role of *Caspase-1* variant in influencing cognitive function through changing tau related-neurodegeneration process, suggesting this genetic locus plays a considerable role in tau related neurodegenerative diseases. The specific mechanism of how the genetic variations in *Caspase-1* influence tau pathology still needs further study in cellular and animal models for the time to come.

## Supplementary Information

**Additional file 1: Table S1**. The correlation between covariates and CSF biomarkers, image data at baseline in a multiple linear regression model. **Table S2**. The correlation between *Caspase-1* rs554344 and longitudinal CSF biomarkers, image data in total non-demented elderly group. **Figure S1**. The results of mediation analyses. We found that *Caspase-1* acting on FDG PET levels did not mediate through CSF t-tau or CSF p-tau.

## Data Availability

The datasets used and/or analyzed during the current study are available from the corresponding author on reasonable request.

## Abbreviations

Aβ: Amyloid-β
AD: Alzheimer’s disease
ADNI: Alzheimer’s Disease Neuroimaging Initiative
CSF: Cerebrospinal fluid
FDG: Fluorodeoxyglucose
HV: Hippocampal volume
IL-1β: Interleukin-1β
MCI: Mild cognitive impairment
MMSE: Mini-Mental State Examination
NC: Normal cognition
NIA-AA: National Institute on Aging and Alzheimer’s Association
PET: Positron-emission tomography
*p-tau*: phosphorylated tau; t-tau: total tau
