# Correction to: *Caspase-1* variant influencing CSF tau and FDG PET levels in non-demented elders from the ADNI cohort

**DOI:** 10.1186/s12883-022-02616-2

**Published:** 2022-03-21

**Authors:** Yi Liu, Meng-Shan Tan, Zuo-Teng Wang, Wei Xu, Lan Tan

**Affiliations:** Department of Neurology, Qingdao Municipal Hospital, Qingdao University, Qingdao, China

**Correction to: BMC Neurol 22, 59 (2022)**

**https://doi.org/10.1186/s12883-022-02582-9**

Following publication of the original article [1], the authors reported an error in Fig. 2 where “CSF p-tau” was written as “CSF t-tau” and “CSF t-tau” was written as “FDG PET”. The correct Fig. 2 is presented below.

**Fig. 2:**
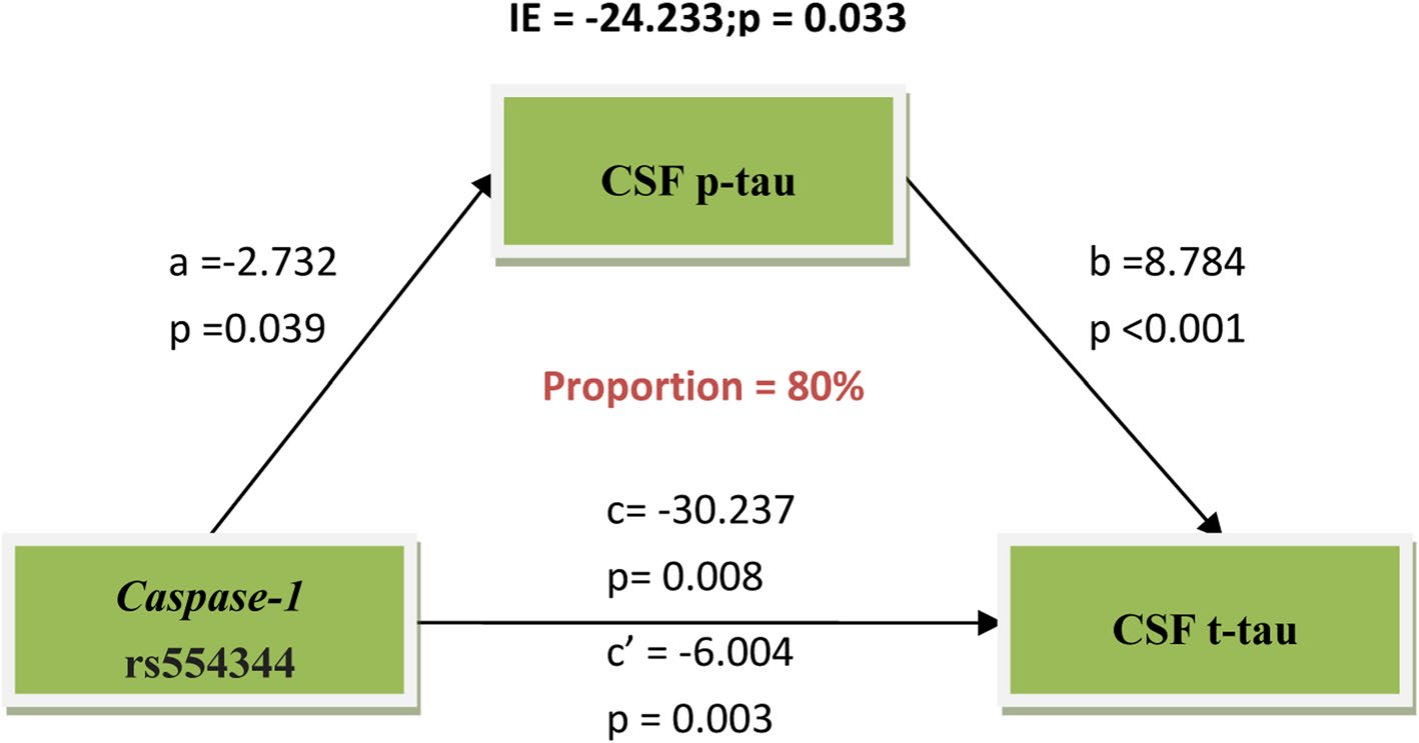
The relationship between *Caspase-1* rs554344 and CSF t-tau levels was mediated by CSF p-tau. The total effect of *Caspase-1* rs554344 on CSF t-tau was estimated and was divided into direct effect and the mediated effect through CSF p-tau. The mediation analysis showed that CSF p-tau significantly and partially mediated the association between *Caspase-1* variant and CSF t-tau levels, accounting for 80% of the total effect

The original article [1] has been updated.
